# Rapid detection of *Mycobacterium tuberculosis* in sputum using CRISPR-Cas12b combined with cross-priming amplification in a single reaction

**DOI:** 10.1128/jcm.00923-23

**Published:** 2023-12-19

**Authors:** Lijun Peng, Tingting Fang, Qingshan Cai, Hao Li, Huanyu Li, Haiqiong Sun, Mingzhi Zhu, Lingshan Dai, Yanqin Shao, Long Cai

**Affiliations:** 1 Clinical Laboratory Center, Affiliated Hangzhou Chest Hospital, Zhejiang University School of Medicine, Hangzhou, Zhejiang, China; 2 Department of Tuberculosis, Affiliated Hangzhou Chest Hospital, Zhejiang University School of Medicine, Hangzhou, China; The University of North Carolina at Chapel Hill School of Medicine, Chapel Hill, North Carolina, USA

**Keywords:** CRISPR, Cas12b, cross-priming amplification, *Mycobacterium tuberculosis*, nucleic acid detection

## Abstract

The diagnosis of tuberculosis (TB) still lacks a rapid, user-friendly method. Recent research has revealed the significant diagnostic potential of the CRISPR system in TB detection. However, most current CRISPR-based diagnostic tests involve a two-step process, including nucleic acid amplification followed by CRISPR-guided sequence-specific detection, which raises concerns about cross-contamination and operational complexity. In this study, we introduced TB One-Pot, which combines CRISPR-Cas12b-mediated trans-cleavage with cross-priming amplification, enabling one-pot detection in a single reaction. TB One-Pot achieves a minimum detection limit of 0.8 copies/μL for H37Rv genomic DNA and a quantification limit of 50 CFU/mL. It exhibited no cross-reactivity with common nontuberculous mycobacteria and respiratory pathogens. TB One-Pot employs real-time fluorescence, enabling the process from nucleic acid extraction to result in reporting in just 80 minutes. It also includes a UV-based visual check, eliminating the need for specialized equipment. A retrospective cohort study evaluated the diagnostic performance of this method using a composite reference standard as the gold standard. TB One-Pot demonstrated a sensitivity of 67.2% and an area under the curve (AUC) of 0.820 in sputum samples, surpassing conventional methods such as culture and acid-fast bacilli (AFB) smear. Comparative analysis with Xpert (sensitivity:64.2%, AUC:0.821) showed no statistically significant difference (*P* > 0.05). TB One-Pot exhibited a specificity of 96.7%, similar to Xpert, culture, and AFB smear (*P* > 0.05). TB One-Pot excels in speed, sensitivity, specificity, and practical requirements. Ongoing refinements aim to improve sensitivity, making it a promising solution for overcoming limitations in molecular TB diagnosis, particularly in resource-constrained settings.

**IMPORTANCE:**

In this study, we successfully established a new One-Pot method, named TB One-Pot, for detecting Mtb in sputum by combining CRISPR-cas12b-mediated trans-cleavage with cross-priming amplification (CPA). Our study evaluated the diagnostic performance of TB One-Pot in clinical sputum samples for tuberculosis. The findings provide evidence for the potential of TB One-Pot as a diagnostic tool for tuberculosis.

## INTRODUCTION

Tuberculosis (TB), caused by *Mycobacterium tuberculosis* (Mtb), remains a significant global infectious disease and a formidable health challenge. In 2021, it was estimated that approximately 10.6 million people worldwide were affected by TB, with 1.6 million deaths attributed to the disease. The TB incidence rate increased by 3.6% compared to 2020 (1). At present, TB clinical diagnosis depends on clinical symptoms, radiological findings, and laboratory tests. The microbiological diagnostic methods for TB mainly involve acid-fast bacilli (AFB) smear microscopy, mycobacterial culture, and molecular detection. AFB smear microscopy, while cost-effective, exhibits lower sensitivity (around 30%), limiting its widespread use in TB diagnosis (2, 3). AFB smear microscopy lacks specificity in distinguishing Mtb from nontuberculous mycobacteria (NTM). While culture is considered a “gold standard” with moderate sensitivity (39%–70%), its utility is limited by infrastructure and lengthy incubation (2–8 weeks) (2–8). Advancements in molecular diagnostics have led to the rapid evolution of TB testing (9), exemplified by Xpert MTB/RIF (Cepheid, USA). This automated, cartridge-based system uses semi-nested real-time Polymerase chain reaction (PCR) targeting the *rpoB* gene, providing Mtb and rifampicin resistance results in just 2 hours (10). Xpert demonstrates solid sensitivity (59%–75%) and high specificity (96%–100%) (4, 11, 12). However, the high equipment and cartridge costs limit Xpert’s accessibility, especially in less-developed countries. Sensitivity and specificity assessment for these three methods follows composite reference standards (CRS). Despite various TB diagnostic methods, only 59% of cases receive microbiological confirmation, per the WHO’s report (13). To achieve the ambitious goal of TB elimination by 2035, there is an urgent need to develop highly sensitive, user-friendly, rapid, and field-applicable diagnostic methods that can be readily implemented in resource-limited settings.

PCR relies on costly thermocyclers and heat-stable DNA polymerases, making it unsuitable for point-of-care testing (POCT). By contrast, isothermal amplification is a straightforward, rapid, and efficient nucleic acid amplification process that can substitute expensive thermocyclers with a water bath, significantly streamlining the nucleic acid amplification workflow. With the emergence of various isothermal polymerases, a range of enzyme-mediated isothermal nucleic acid amplification techniques have surfaced, including loop-mediated isothermal amplification (LAMP) (14), recombinase polymerase amplification (RPA), and cross-priming amplification (CPA) (12, 15). CPA relies on a chain-replacing DNA polymerase and is capable of exponential amplification of DNA target sequences, guided by multiple cross-linked primers (Ustar Biotechnologies, Hangzhou, China). This approach exhibits a high level of specificity, sensitivity, and rapidity in diagnosing TB while being much more cost-effective than Xpert MTB/RIF (12, 16). Although isothermal amplification has overcome temperature control challenges, it still faces limitations related to false positives, nucleic acid sequence compatibility, and signal amplification capacity. Fortunately, by integrating isothermal amplification with the Clustered Regularly Interspaced Short Palindromic Repeat (CRISPR) enzymes to achieve cascade bioconversion, these limitations can be effectively addressed (15).

CRISPR system consisting of CRISPR-associated (Cas) proteins and CRISPR RNA (crRNA), serves as an adaptive immune defense system in bacteria and archaea, conferring resistance against invasive mobile genetic elements (17). In the CRISPR system, Cas proteins, guided by crRNAs, recognize target nucleic acids, forming ternary complexes that facilitate trans-cleavage of fluorophore-quencher (FQ)-labeled single-stranded DNAs or RNAs (FQ reporters) (18). This cleavage generates detectable fluorescent signals. The highly efficient trans-cleavage acts as a superior signal amplification method, elevating sensitivity by approximately two to three orders of magnitude (15). The combination of the CRISPR system with isothermal methods for pathogen detection has resulted in the development of various CRISPR-based nucleic acid detection techniques (18, 19). These methods offer rapid, highly sensitive, specific, convenient, and cost-effective diagnostic solutions, garnering significant attention in clinical diagnosis. Notable examples include HOLMES (1-hour low-cost multipurpose highly efficient system) based on CRISPR/Cas12a (20), as well as SHERLOCK (specific high sensitivity enzymatic reporter unlocking) utilizing CRISPR/Cas13a (19). These approaches enable the rapid detection of a wide range of DNA and RNA pathogens while achieving single-base mismatch specificity and attomolar sensitivity.

Current CRISPR-based diagnostic methods typically require a two-step process involving nucleic acid amplification followed by CRISPR-guided sequence-specific detection. However, this approach raises concerns about the potential for cross-contamination and operational complexity. To our knowledge, current CRISPR-based TB detection systems all involve a two-step process (4–8, 21–24). To address these limitations, we have developed an innovative TB detection method, which we have named TB One-Pot. TB One-Pot is a CPA-CRISPR-based nucleic acid detection platform that integrates target preamplification with CRISPR/Cas12b-based detection into a single reaction mixture, conducted at a constant temperature. It can accurately identify Mtb using nucleic acids from pure cultures or clinical samples with a single fluid-handling step. We conducted a retrospective cohort study involving 293 patients to evaluate the diagnostic capability of TB One-Pot in clinical sputum samples for TB.

## MATERIALS AND METHODS

### Preparation of DNA templates

**Fig 1:**
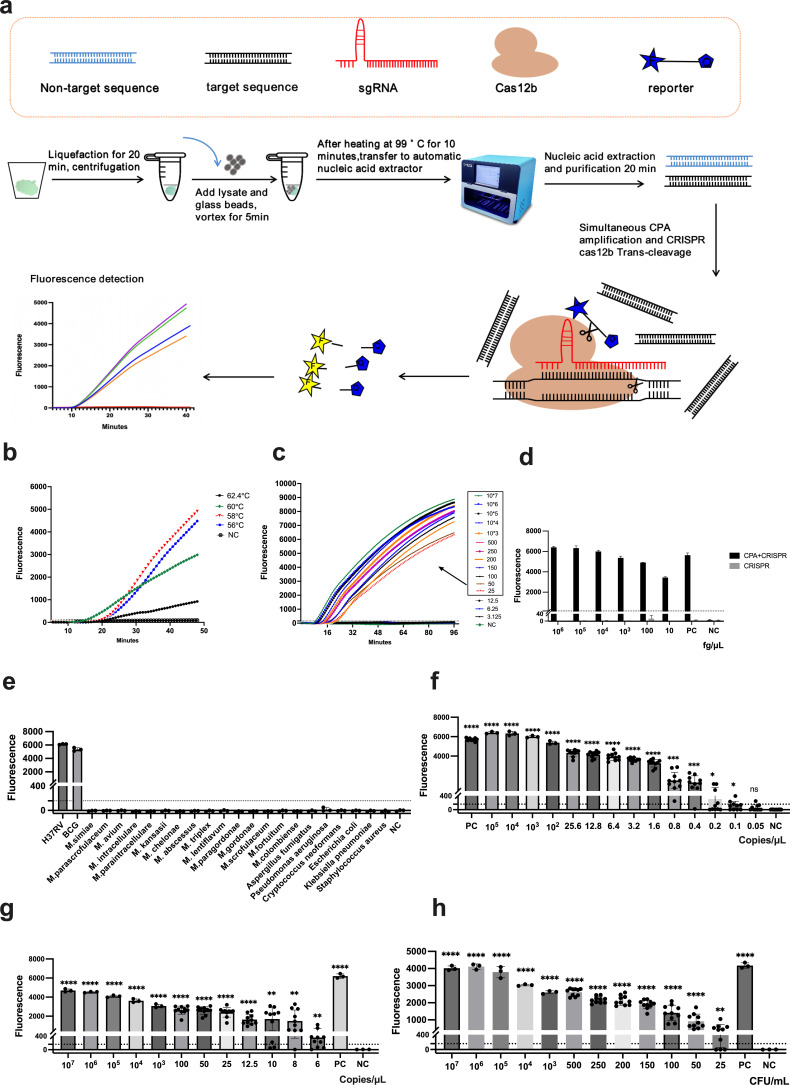
Description and evaluation of the TB One-Pot detection platform. (**a**) TB One-Pot workflow: A 5 µL DNA template, obtained through glass bead physical disruption and automated nucleic acid extraction, is mixed with the CPA amplification and CRISPR systems. Cas proteins guided by gRNAs recognize the target nucleic acids, forming ternary complexes that trans-cleave fluorophore-quencher (FQ)-labeled ssDNA. The FAM signal was monitored at 58°C for 32 minutes in the fluorescence PCR instrument. (**b**) Optimization of TB One-Pot detection temperature: TB One-Pot was incubated at various temperatures (56°C, 58°C, 60°C, and 62.4°C) for 48 minutes, with fluorescence signals recorded every 48 seconds. The input was 10 fg H37Rv genomic DNA. The colored line represents the mean value of three replicates. (**c**) Determination of TB One-Pot reaction time: Gradient samples were monitored for FAM fluorescence signals at 58°C for 96 minutes (*n* = 1). (**d**) TB One-Pot (CPA + CRISPR) vs CRISPR reaction without CPA: The input was serially diluted H37Rv genomic DNA (*n* = 3, means ± SD). (**e**) Determination of species specificity of TB One-Pot: Testing with DNA from H37Rv, BCG, 15 NTM strains, and six common respiratory pathogen strains (*n* = 3, means ± SD). (**f–h**) Determination of TB One-Pot limit of detection (LOD). (**f**) LOD for H37Rv genomic DNA,10 repetitions for concentrations up to 25.6 copies/μL, and three repetitions for higher concentrations. (**g**) LOD for pUC-IS*6110* plasmid DNA,10 repetitions for concentrations up to 100 copies/μL, and three repetitions for other concentrations. (**h**) LOD determination using samples with concentrations ranging from 10^7^ to 25 CFU/mL spiked into negative sputum samples. Those with up to 500 CFU/mL were tested 10 times, while the rest underwent three repetitions. The LOD was defined as the lowest point with a 100% detection rate, with 1 ng/µL H37Rv genomic DNA as the positive control (PC) and RNase-free water as the negative control (NC).

DNA was extracted from 1 to 3 mL of sputum samples. The sputum was mixed with N-acetyl-L-cysteine (NALC)-NaOH solution in a 2:1 ratio and incubated at room temperature for 20 minutes. Following this, 1 mL of the processed sputum samples was transferred to sterile and nuclease-free 1.5 mL tubes. After centrifugation at 12,000 × *g* for 5 minutes, the pellet was resuspended in 50 µL of lysis buffer. Glass beads with a diameter of 0.1–0.2 mm (25 mg) were added to each tube. The mixture was then vortexed at a speed of 3,000 rpm for 5 minutes using a Crystal vortex mixer. Subsequently, the tubes were heated at 99°C for 10 minutes. The liquid from the tubes was transferred to the extraction well of an automated nucleic acid extraction system and processed according to the protocol, taking approximately 20 minutes. Finally, 5 µL of the eluted nucleic acid was collected as the template for subsequent experiments (Fig. 1a).

Isolates were selected from solid cultures, and glass beads (25 mg, 0.1–0.2 mm in diameter) along with 50 µL of lysis buffer were added to each tube. The vortex mixer was utilized at a speed of 3,000 rpm for 5 minutes to disrupt the bacterial cell walls. The tubes were then heated at 99°C for 10 minutes and subsequently centrifuged at 12,000 × *g* for 2 minutes. Finally, 5 µL of the supernatant was collected as the template for further experiments. The DNA concentration of the isolated strains was determined using a Nanodrop 2000 spectrophotometer (Thermo Fisher, USA) or a Qubit (Thermo Fisher, USA）.

### Establishment and optimization of the TB One-Pot detection platform

Establishment of the TB One-Pot detection platform: The IS*6110* insertion sequence, exclusive to *Mtb*, exists as multiple copies within the H37Rv genome but is absent in other strains. Specifically, there are 16 complete copies of IS*6110* in H37Rv (6). In this study, we selected the IS*6110* fragment as the target gene for TB One-Pot detection. The TB One-Pot detection comprises the target nucleic acid template and components A and B. Component A includes a 10 × reaction buffer, CRISPR Cas12b (Ustar Biotechnologies, Hangzhou, China), trRNA, crRNA, and RNase-free water. Component B consists of five CPA primers, two ssDNA-FQ reporter genes, and a 1 × Core Reaction Buffer. The primers, ssDNA fluorescent probes, and gRNA were synthesized and purified at HPLC grade (Hangzhou Youkang Biotechnology, Zhejiang, China). Component A can be pre-incubated at 48°C for 30 minutes and stored at −20°C for 2 weeks. Thus, we can add 5 µL of Component A, 30 µL of Component B, and 5 µL of the template simultaneously, resulting in a 40 µL reaction volume. Fluorescence was measured using a Bio-Rad CFX96 deep well real-time system (Bio-Rad Laboratories, USA) at a constant temperature, with readings taken every 48 seconds.

Optimization of TB One-Pot detection temperature: The TB One-Pot detection, utilizing a target template of 10 fg of H37Rv DNA, was incubated at various temperatures (56°C, 58°C, 60°C, and 62.4°C). Fluorescence values were recorded every 48 seconds during a total monitoring duration of 48 minutes, and the optimal detection temperature was determined based on the recorded fluorescence values.

Determination of TB One-Pot reaction time: Samples with gradient concentrations ranging from 10^7^ CFU/mL to 3.125 CFU/mL were incubated at the optimal temperature. Fluorescence values were measured every 48 seconds, with a total monitoring duration of 96 minutes, and the optimal reaction time was determined based on the positive results.

### Specificity assessment and limit of detection

Genomic DNA was extracted from 21 bacterial strains and two fungal strains to confirm the specificity of the TB One-Pot system. This set of strains included eight strains stored in our laboratory: Mtb H37Rv (ATCC 25293), *M. bovis* bacillus Calmette-Guerin (BCG), *Pseudomonas aeruginosa* (ATCC 9027), *Escherichia coli* (ATCC 25922), *Klebsiella pneumoniae* (ATCC 700603), *Staphylococcus aureus* (ATCC 29213), *Aspergillus fumigatus*, and *Cryptococcus neoformans*. In addition, bacterial DNA from 15 NTM strains, accurately identified through whole-genome sequencing (WGS), was included. These NTM strains comprised *M. simiae, M. parascrofulaceum, M. avium, M. intracellulare, M. paraintracellulare, M. kansasii, M. chelonae, M. abscessus, M. triplex, M. lentiflavum, M. paragordonae, M. gordonae, M. scrofulaceum, M. fortuitum,* and *M. colombiense*. Under the optimized reaction conditions, the species specificity of the TB One-Pot was assessed. The specificity test was performed in triplicate, with a detection concentration of 1 ng/µL for all strains.

We determined the limit of detection (LOD) for both H37Rv genomic DNA and plasmid DNA and quantified the CFU LOD. The target segment of IS*6110* was cloned into the pUC57 plasmid, generating a single-copy pUC-IS*6110* plasmid (GenScript, China) to be used as the template. The concentration of H37Rv genomic and plasmid DNA was measured using a Qubit, and copy numbers were calculated using the following formula: (6.02 × 10^23^) × (ng/μL × 10^−9^) / (DNA length ×660) =copies/μL. For the LOD of H37Rv genomic DNA, we performed gradient dilutions from 10^5^ copies/μL to 0.05 copies/μL, conducting 10 replicates for concentrations ≤ 25.6 copies/μL, and three replicates for other concentrations. The pUC-IS*6110* plasmid was diluted in RNase-free water, resulting in dilutions ranging from 10^7^ copies/μL to six copies/μL. Ten replicates were performed for concentrations ≤ 100 copies/μL, while three replicates were conducted for other concentrations. For CFU quantification, the viable bacterial suspension of the reference strain H37Rv of Mtb was subjected to continuous dilution and inoculated onto a solid culture medium. The concentration of live bacteria (CFU/mL) was calculated based on the colony count. In this study, a suspension of live bacteria with an approximate concentration of 10^8^ CFU/mL was prepared and subjected to repeated experiments. The range of concentrations spanned from 10^7^ CFU/mL to 25 CFU/mL, with subsequent addition to negative sputum samples. The TB One-Pot assay was evaluated by determining the percentage of successful TB detections at each input CFU concentration in sputum. Samples with concentrations ≤500 CFU/mL were tested with 10 replicates, while other concentrations were tested with three replicates. The LOD was determined as the lowest point with a 100% detection rate.

### Study participants

We conducted a retrospective study to collect sputum samples and medical records of suspected pulmonary TB patients who sought treatment at Affiliated Hangzhou Chest Hospital, Zhejiang University School of Medicine, between November 2022 and March 2023. Notably, this hospital serves as the Zhejiang Provincial Tuberculosis Diagnosis and Treatment Center. Sample selection for this study was performed using a single-blinded, randomized approach. Inclusion criteria included the following: Eligible participants were those who underwent simultaneous testing using three diagnostic methods, namely AFB smear microscopy, culture, and GeneXpert MTB/RIF. In addition, a minimum volume of 1 mL of sample was required to ensure feasibility for TB One-Pot detection. Patients with uncertain final diagnoses, duplicate samples, or insufficient sample volume were excluded from the study.

### Patient classification

Following the China Clinical Treatment Guide for Tuberculosis (25) and the Tuberculosis Treatment Guidelines (26), clinicians classify patients into two groups using the CRS, which includes clinical symptoms, physical signs, laboratory findings, radiological images, and follow-up data: (1) TB group: all TB patients exhibit clinical symptoms and chest imaging features associated with TB. Patients with positive Mtb cultures or positive Xpert results are categorized as microbiologically confirmed TB cases. In addition, patients without microbiological evidence were clinically diagnosed with active Mtb infection based on clinical symptoms, positive QuantiFERON TB-GOLD (QFT) or T-SPOT test outcomes, and subsequent treatment response after a 1-month follow-up period (evidenced by symptom improvement and resolution) (2). Non-TB group: patients definitively diagnosed with diseases other than TB.

### AFB smear, culture, and molecular detection

Using Ziehl-Neelsen acid-fast staining reagents, AFB smear microscopy was performed on sputum samples. Both Mtb and NTM appear AFB positive on the slides due to their bacterial characteristics. A positive AFB result requires confirmation with other test results to distinguish between Mtb and NTM. Approximately 2 mL of sputum samples was decontaminated using N-acetyl-L-cysteine-sodium hydroxide (NALC-NaOH). After neutralization with sterile saline phosphate buffer (PBS, pH 6.8) and centrifugation, the pellet was inoculated into a liquid medium using the MGIT 960 system. All positive cultures were subsequently confirmed using the CapitalBio Mycobacterium RT-PCR Detection Kit (CapitalBio Technology, China). The CapitalBio Mycobacterium RT-PCR Detection Kit allows for the simultaneous screening of Mtb and NTM infections. Identification as Mtb indicates a positive culture, while identification as NTM or negative is considered a negative culture (27). Conducting molecular detection with Xpert, following the manufacturer’s instructions. The system automatically generated Mtb detection results and assessed rifampicin resistance status within 2 hours.

### Data processing and statistical analysis

Statistical analysis was conducted using MedCalc Statistical Software to determine the sensitivity, specificity, positive predictive value (PPV), negative predictive value (NPV), and area under the curve (AUC) for the different diagnostic methods. The cutoff values were determined by identifying the point on the receiver operating characteristic (ROC) curve with the maximum Youden index, which represents the optimal balance between sensitivity and specificity. We compared continuous variables using *t*-tests, and categorical variables using chi-squared tests, and employed Z-tests for AUC comparisons. All statistical tests were two-tailed, and a significance level of *P* < 0.05 was considered statistically significant. The Venn diagram was generated using an online tool (https://hiplot.com.cn), and data visualization was performed using GraphPad Prism.

## RESULTS

### Establishment of TB One-Pot detection

The TB One-Pot detection process is illustrated in Fig. 1a. Initially, we conducted a compatibility test to assess the performance of two enzymes within a single reaction buffer. As shown in Fig. S1, distinct fluorescence signals indicative of CPA amplification and cas12b-mediated trans-cleavage enzyme activity were observed in positive samples, indicating the effective functionality of CPA and Cas12b within the same reaction system. Subsequently, the reaction system was subjected to varying temperatures (56°C, 58°C, 60°C, and 62.4°C) to detect H37Rv genomic DNA at a concentration of 10 fg. Notably, the fluorescence signal generated by cas12b-mediated trans-cleavage enzyme activity was found to be the strongest at 58°C (Fig. 1b). Furthermore, all detectable positive results were observed within 32 minutes (Fig. 1c), and extending the reaction to 96 minutes did not yield any additional positive results. Therefore, the final optimized reaction conditions were determined as follows: 58°C for 32 minutes. Moreover, CPA amplification proved to be essential for TB One-Pot detection. We observed that the inclusion of up to 1 ng/µL of H37Rv genomic DNA and 10^7^ copies/μL of plasmid directly into the CRISPR reaction mixture (comprising Cas12b, trRNA, crRNA, ssDNA-FQ reporter probe, and 1 × core reaction buffer) did not activate Cas12b’s trans-cleavage activity within 32 minutes. However, upon further increasing the plasmid concentration to 10^10^ copies/μL, we successfully activated Cas12b’s trans-cleavage activity, resulting in detectable fluorescence signals (Fig. 1d; Fig. S2).

Strain specificity was determined by utilizing genomic DNA samples from clinical isolates obtained from our laboratory, as well as standard bacterial strains. The fluorescence values in the reaction tubes containing DNA samples from 15 different NTM strains (*M. simiae, M. parascrofulaceum, M. avium, M. intracellulare, M. paraintracellulare, M. kansasii, M. chelonae, M. abscessus, M. triplex, M. lentiflavum, M. paragordonae, M. gordonae, M. scrofulaceum, M. fortuitum,* and *M. colombiense*) and six common respiratory pathogen strains (*Aspergillus fumigatus, Cryptococcus neoformans, Pseudomonas aeruginosa, Escherichia coli, Klebsiella pneumoniae,* and *Staphylococcus aureus*) exhibited low fluorescence values (<threshold of 157.7, determined based on the highest Youden’s Index point on the ROC curve constructed using positive patient samples and negative samples, including negative patients and all negative controls used in this study). By contrast, H37Rv and BCG showed high fluorescence values (>5,000) (Fig. 1e). TB One-Pot demonstrated excellent strain specificity as it did not exhibit any cross-reactivity with the aforementioned common NTM strains and respiratory pathogens.

This study assessed the LOD using three distinct nucleic acid targets. When employing H37Rv genomic DNA, TB One-Pot achieved a 100% positive detection rate at concentrations equal to or greater than 0.8 copies/μL, establishing a LOD of 0.8 copies/μL of genomic DNA (Fig. 1f). In the case of single-copy plasmid DNA, TB One-Pot exhibited a 100% positive detection rate at concentrations of 12.5 copies/μL or higher, resulting in a LOD of 12.5 copies/μL (Fig. 1g). Furthermore, when subjecting TB One-Pot to simulated sputum samples containing H37Rv, a positive detection rate of 100% was observed at concentrations equal to or greater than 50 CFU/mL (Fig. 1h). Consequently, the LOD for this method was conclusively determined to be 50 CFU/mL.

### Application of TB One-Pot in clinical sputum samples

**Fig 2:**
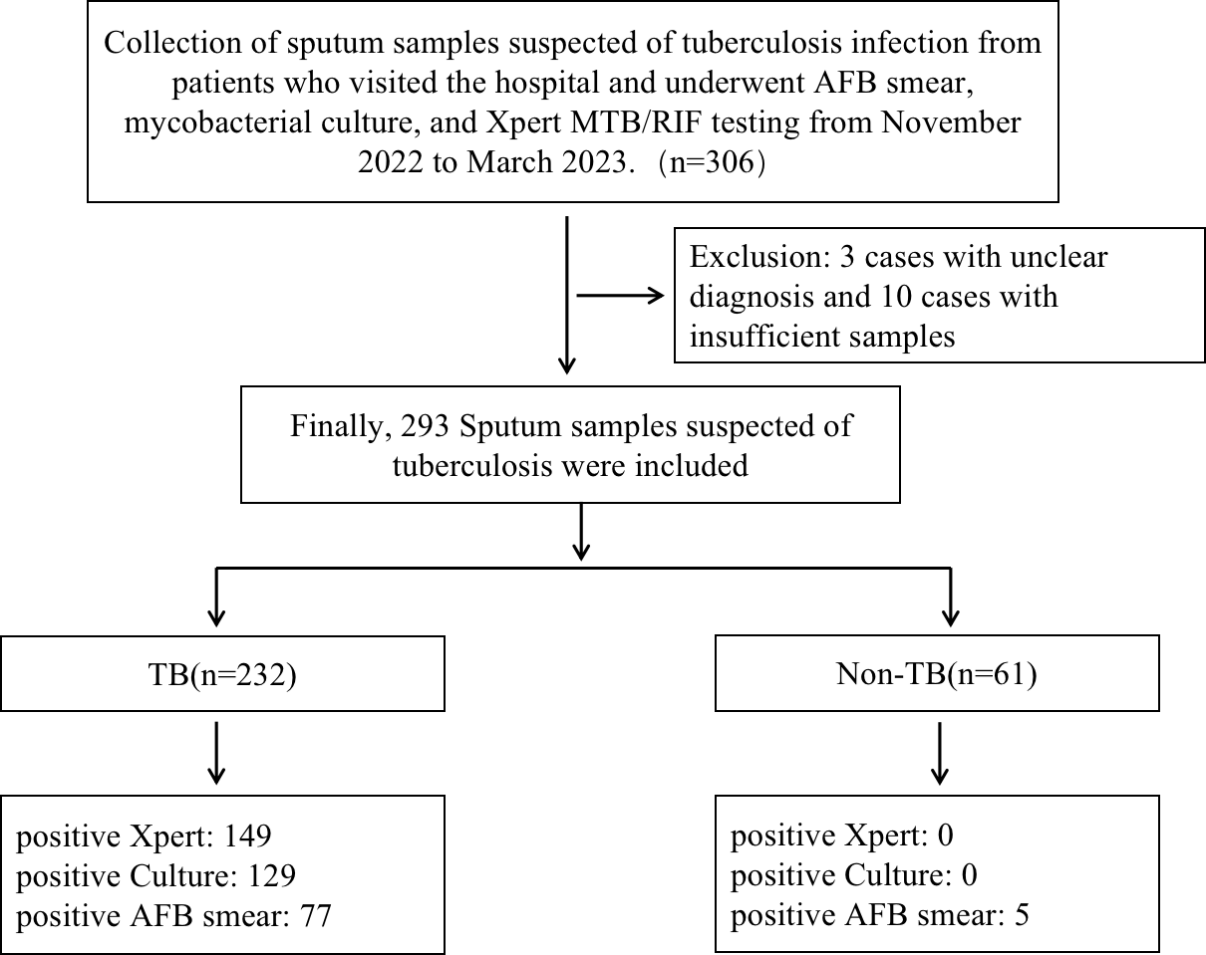
Sample inclusion flowchart.

A total of 306 sputum samples were collected from individuals suspected of having active pulmonary TB. In total, 13 cases were excluded due to indeterminate final diagnoses or inadequate sample volumes, resulting in a final inclusion of 293 cases, with 232 cases in the TB group and 61 cases in the non-TB group (Fig. 2). Within the TB group, 149 cases tested positive by Xpert, 129 cases showed positive results in Mtb culture, and 77 cases exhibited AFB smear positivity. By contrast, all 61 cases in the non-TB group tested negative for both Xpert and Mtb culture. Notably, among the non-TB samples, five were AFB smear positive and clinically diagnosed as NTM cases. The study population consisted of 230 male patients (78.5%) and 63 female patients (21.5%). The median age of the study cohort was 60 years, ranging from 8 to 91 years. TB patients [median 61.5 (33.3–74)] were relatively older compared to non-TB patients [median 52.0 (8–67)], and there was a higher proportion of male individuals in the TB group.

The sensitivity, specificity, PPV, NPV, and AUC of TB One-Pot for the diagnosis of TB were calculated as 67.2% (95% CI 60.8%–73.2%), 96.7% (95% CI 88.7%–99.6%), 98.7% (95% CI 95.2%–99.7%), 43.7% (95% CI 39.1%–48.4%), and 0.820 (0.771–0.862) respectively. TB One-Pot demonstrated slightly higher sensitivity and NPV compared to Xpert, while the specificity, PPV, and AUC were slightly lower than Xpert. However, no statistically significant differences were observed between the two methods for these performance indicators (*P* > 0.05). Notably, the sensitivity and AUC of TB One-Pot were significantly higher than those of culture and AFB smear (*P* < 0.05) (Table 1).

**TABLE 1:** Diagnostic performance of several tests in sputum samples

Test	CRS diagnosis		Sensitivity	Specificity	PPV	NPV	AUC
	TB (*n* = 232)	Non-TB (*n* = 61)	% (95%CI)	% (95%CI)	% (95%CI)	% (95%CI)	(95%CI)
TB One-Pot			67.2 (60.8–73.2)	96.7 (88.7–99.6)	98.7 (95.2–99.7)	43.7 (39.1–48.4)	0.820 (0.771–0.862)
Positive	156	2					
Negative	76	59					
Xpert			64.2 (57.7–70.4) ^*a*^	100.0 (94.1–100.0) ^*a*^	100.0 (100.0–100.0) ^*a*^	42.4 (38.2–46.6) ^*a*^	0.821 (0.772–0.863) ^*a*^
Positive	149	0					
Negative	83	61					
Culture			55.6 (49.0–62.1) ^*b*^	100.0 (94.1–100.0) ^*a*^	100.0 (100.0–100.0) ^*a*^	37.2 (33.9–40.6) ^*a*^	0.778 (0.726–0.824) ^*b*^
Positive	129	0					
Negative	103	61					
AFB			33.2 (27.2–39.7) ^*b*^	91.8 (81.9–97.3) ^*a*^	93.9 (86.7–97.3) ^*a*^	26.5 (24.3–28.9) ^*b*^	0.625 (0.567–0.681) ^*b*^
Positive	77	5					
Negative	155	56					

^*a*^ TB One-Pot showed no statistically significant difference (*P* > 0.05) when compared to Xpert, culture, and AFB smear.

^*b*^ TB One-Pot showed a statistically significant difference (*P* < 0.05) when compared to Xpert, culture, and AFB smear; CI, confidence interval; PPV, positive predictive value; NPV, negative predictive value; AUC, area under the curve; CRS, composite reference standard.

**Fig 3:**
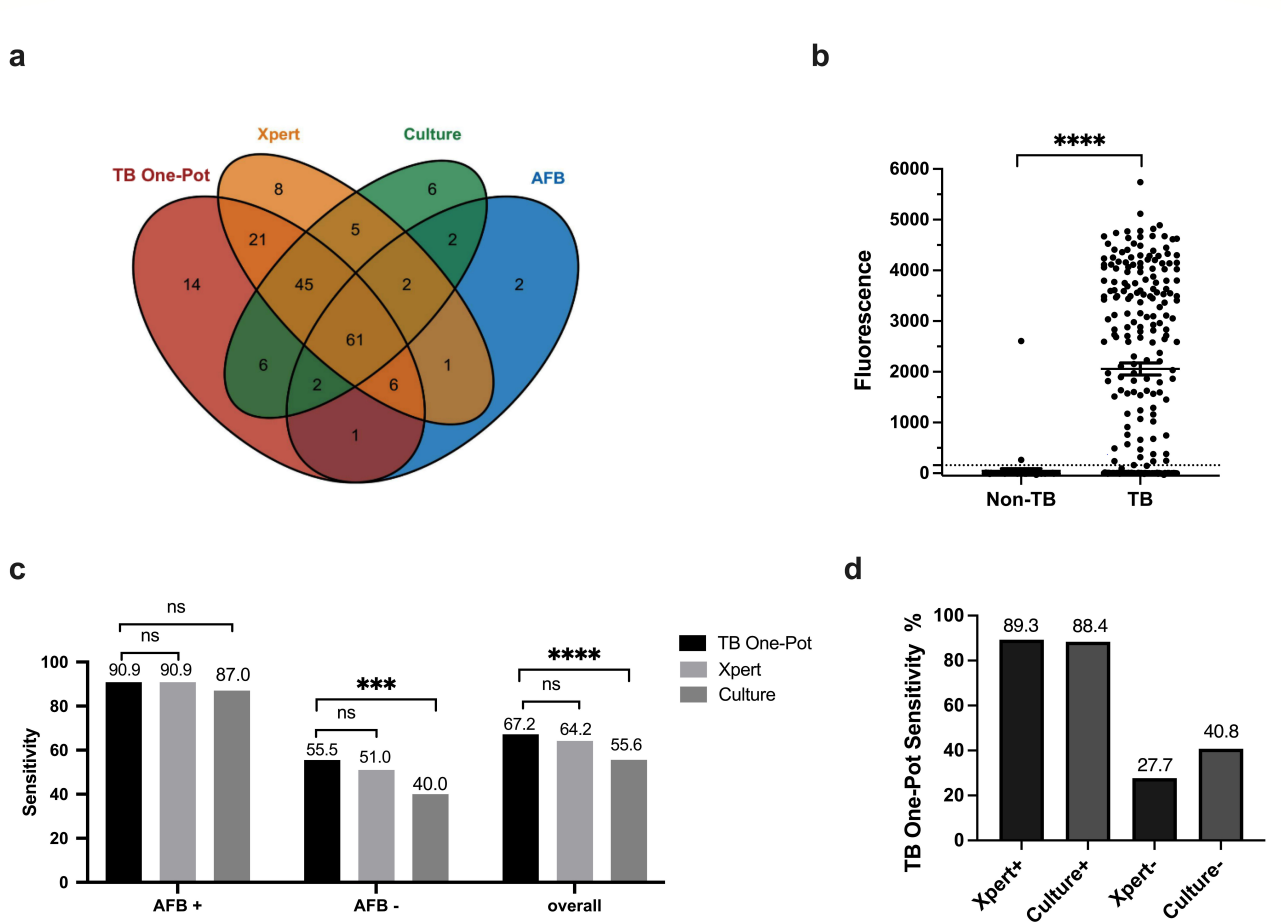
Performance of TB One-Pot in sputum samples. (**a**) Venn diagram showing TB One-Pot, Xpert, mycobacterial culture, and AFB smear results in TB samples. (**b**) TB One-Pot was performed with sputum DNA from active TB patients (*n* = 232) and non-TB patients (*n* = 61). The dotted line represents the cutoff value at 157.7, and the black bars represent the mean with standard error of the mean (SEM). Unpaired *t*-test, ∗∗∗∗*P* < 0.0001. (**c**) A comparison of TB One-Pot, Xpert assays, and M.tb culture in AFB smear-positive and AFB smear-negative patients. Chi-square test, ∗∗∗ *P* < 0.001, ∗∗∗∗*P* < 0.0001. (**d**) Detection rates of TB One-Pot in TB patients with positive and negative Xpert and mycobacterial culture results.

The Venn diagram illustrating the positive samples from TB patients, using AFB smear, culture, Xpert, and TB One-Pot, revealed the overlapping distribution of these methods. When considering the detection of positive cases using a single method, TB One-Pot identified 14 cases, whereas Xpert, culture, and AFB smear detected 8, 6, and 2 cases, respectively (Fig. 3a). Moreover, the fluorescence values detected in the TB group were significantly higher than those in the non-TB group (*P* < 0.0001) (Fig. 3b). The detection rates of TB One-Pot, Xpert, and *Mtb* culture were comparable among AFB smear-positive TB patients (90.9%, 90.9%, and 87.0%, respectively) (*P* > 0.05). In AFB smear-negative TB patients, the sensitivity of TB One-Pot (55.5%) was similar to that of Xpert (51.0%) (*P* > 0.05), but significantly higher than that of culture (40.0%) (*P* < 0.05) (Fig. 3c). Furthermore, in Xpert-positive and culture-positive samples, TB One-Pot exhibited sensitivities of 89.3% and 88.4%, respectively. In Xpert-negative and culture-negative samples, the sensitivities of TB One-Pot were 27.7% and 40.8%, respectively (Fig. 3d).

## DISCUSSION

There have been nine published studies utilizing CRISPR Cas protein trans-cleavage activity for TB detection (4–8, 21–24). These studies employed a two-step approach, involving the pre-amplification of the target sequence using techniques such as PCR, LAMP, or RPA, followed by the transfer of the amplified products to the CRISPR reaction system. However, this two-step process is laborious and carries a higher risk of sample cross-contamination. Cas12a and Cas13a exhibit relatively low tolerance to high temperatures. Hence, these methods may encounter reduced reaction efficiency or premature target amplification at room temperature, limiting quantification accuracy. In our study, we utilized CRISPR Cas12b protein derived from *Alicyclobacillus acidophilus*, which exhibits maximum cleavage activity between the temperatures of 31°C and 59°C. This Cas12b protein demonstrates remarkable characteristics, including high specificity, stability, and minimal off-target effects (28–30). Therefore, AapCas12b can be used in isothermal nucleic acid amplification methods like LAMP and CPA, which operate between 55°C and 65°C (28–30). Experimental results confirmed the compatibility and effectiveness of the CRISPR Cas12b system and CPA isothermal amplification system at a temperature of 58°C. We successfully developed the TB One-Pot, enabling the entire process from nucleic acid extraction to result reporting to be completed in just 80 minutes.

In this study, we made an intriguing observation that Cas12b exhibits characteristics similar to Cas13a. It functions suboptimally without nucleic acid amplification and only becomes activated at elevated target concentrations (19). Sam et al.’s TB-QUICK previously demonstrated its failure to activate Cas12b, even with a 0.3 nM plasmid (6). Notably, even with a genomic DNA concentration of 1 ng/µL (Fig. 1d) or when using plasmids at 10^7^ copies/μL, Cas12b’s trans-cleavage activity could not be directly activated. It was only when we increased the plasmid concentration to 10^10^ copies/μL that we successfully activated Cas12b’s trans-cleavage activity and detected positive fluorescence signals (Fig. S2). Therefore, CPA amplification is a crucial step for TB One-Pot detection.

This study revealed that CPA amplification exhibited low non-specific amplification signals in the negative control (RNase-free water) but did not show any Cas enzyme cleavage signals (Fig. S1). Similarly, during the testing of clinical samples, some negative samples exhibited non-specific amplification signals, but no Cas enzyme cleavage signals were detected. These findings indicate that the inclusion of cas12b effectively mitigates the limitations of non-specific amplification in the CPA method. Consequently, all reported fluorescence values in this study are based on the FAM signals generated through cas12b cleavage. TB One-Pot demonstrates excellent species specificity, as it does not display cross-reactivity with other NTM strains or common respiratory pathogens, except for Mtb and BCG.

TB One-Pot has a competitive LOD. When using H37Rv genomic DNA, TB One-Pot achieved a LOD of 0.8 copies/μL (Fig. 1f). This LOD surpasses that of Huang et al.’s CRISPR-TB method (0.25 copies/μL) (5) but remains below the typical range of LODs reported in various CRISPR-based TB detection studies (1–298 copies/μL) (4–8, 21–24). Considering the variation in the number of IS*6110* copies in different Mtb complex strains’ genomes, related to strain evolution (31), we employed a single-copy plasmid template by cloning the IS*6110* target segment into the pUC57 plasmid. Using this single-copy plasmid DNA, TB One-Pot achieved a LOD of 12.5 copies/μL (Fig. 1g). Analysis using sputum samples spiked with a known quantity of Mtb CFU predicted a clinical LOD of 50 CFU/mL, which is lower than that of Xpert (131 CFU/mL) (32) but higher than Xpert Ultra (15.6 CFU/mL) (33). These LOD values are comparable to those reported by Ai et al. (4) for TB detection using CRISPR/Cas12a.

In our method, the CPA amplification and Cas enzyme cleavage occur simultaneously within a single reaction tube. The isothermal amplification enables exponential amplification of the nucleic acid fragments, while the Cas enzyme cleavage further amplifies the signal. Through two consecutive amplification steps, positive results can be rapidly detected. In nucleic acid samples extracted from sputum samples containing over 500 CFU/mL of Mtb, a positive result can be reported after 16 minutes, significantly reducing the reaction time compared to fluorescence PCR methods (Fig. S3). Despite the use of a fluorescence PCR instrument in this method, TB One-Pot requires simple equipment (such as a water bath) and can be visually assessed under ultraviolet light (Fig. S4). The accuracy of visual assessment needs to be further investigated through comparative studies.

In this study, the sensitivity of TB One-Pot (67.2%) was found to be higher than that of Xpert (64.2%), culture (55.6%), and AFB smear (33.2%). In AFB-negative TB patients, TB One-Pot exhibited a sensitivity of 55.5%, which was slightly higher than Xpert (51.0%) and significantly superior to culture (40.0%) (Fig. 3c). Therefore, TB One-Pot serves as a valuable complement to traditional diagnostic methods (Fig. 3d). While TB One-Pot’s LOD is lower than in the majority of CRISPR-based TB studies, its diagnostic sensitivity (67.2%) falls below the reported range of 78.4% to 100% in previous CRISPR-based TB studies (4–8, 21–24). Several potential factors might explain this difference. First, it could be attributed to the use of pre-extracted and quantified nucleic acids for LOD testing, whereas clinical samples require nucleic acid extraction, which can introduce losses and reaction inhibitors. Second, the variation in study populations is another factor. In our study, the sensitivity of Xpert (64.2%) was lower compared to most CRISPR-based TB studies (66.4%–100%). This difference may be because our study was conducted at the Zhejiang Provincial TB Diagnosis and Treatment Center, where we receive challenging cases from across Zhejiang, including those without microbiological evidence. Patients with a high bacterial load are more likely to be diagnosed at primary healthcare facilities, so those seeking treatment at our facility may have paucibacillary TB. Lastly, although we optimized the reaction conditions for CPA amplification and Cas12b detection, there is still room for improvement in the TB One-Pot method. Further enhancements may be achieved by adjusting ion concentrations and pH, which could lead to better performance. Furthermore, considering that this study only used a single target gene, IS*6110*, for detecting Mtb, it is possible that certain strains may lack this gene, leading to false-negative results. To address this potential limitation and improve sensitivity, we aim to explore a dual-target nucleic acid amplification strategy, similar to the approach used in Xpert Ultra.

The specificity of TB One-Pot for diagnosing TB is 96.7%, which is consistent with the reported range of 94.1% to 100% in previous studies (4–8, 21–24). In this study, among nine NTM samples, eight tested negative using the TB One-Pot method, while one showed a positive result, possibly indicating a co-infection of NTM and Mtb (Tables S1 and S2). Among the 61 patients classified as non-TB infections based on CRS, the TB One-Pot method yielded two positive results. These samples included one patient with malignancy and another with NTM infection. However, subsequent follow-up and targeted sequencing validated the presence of Mtb in these samples, albeit at a low abundance (Table S2). Due to the insufficient sensitivity of existing diagnostic methods such as Xpert, clinical false negatives can occur. Xpert Ultra has shown an approximately 10-fold increase in sensitivity compared to Xpert, but it has not been implemented in the hospital where this study was conducted.

In our study, the sensitivity of TB diagnosis using CPA combined with CRISPR-Cas12b was higher than the first-generation paper strip-based EasyNAT TB test developed by Ustar Biotechnologies (67.2% vs 50.6%), which relies solely on CPA (16). Subsequently, a second-generation assay, EasyNAT MTC, was developed, allowing for rapid TB detection in under 2 hours. This method, similar to Xpert, involves minimal manual sample handling steps, utilizing pre-loaded reagents in a single cartridge to perform DNA extraction, purification, target gene amplification, and detection in three separate chambers within the same cartridge. EasyNAT MTC detected more pulmonary TB patients than Xpert (72.19% vs 61.54%) but with lower specificity (95.00% vs 98.75%) (12). Therefore, further enhancing sensitivity and specificity by combining CPA with CRISPR is feasible. Given the simplicity of the TB One-Pot method, it is possible to conduct the entire testing process at the point of care by integrating it into a compact desktop device, enabling a sample-in-result-out assay. To facilitate the use of TB One-Pot in resource-limited settings, we are currently in the process of integrating this system with the hardware of EasyNAT MTC. In addition, the development of lateral flow strips in the future is also being explored as a potential solution.

This study presents a new TB detection method, priced at $1.4 per test, which is more convenient and cost-effective than some previous TB tests (Table 2). TB One-Pot, as a proof-of-concept technology, can be adapted for the detection of other pathogens like malaria by customizing primers and gRNA. In addition, it has the potential for detecting drug-resistant TB, given that TB drug resistance is associated with mutations in chromosomal genes and drug targets (e.g., *inhA*, *rpoB*) (34, 35).

**TABLE 2:** Comparison of our method with several *Mycobacterium tuberculosis* detection methods^*a*^

No.	Cas enzyme	Molecular diagnostics	Amplification methods	Targets	Detection methods	Detection limit	Cost per test ($)	Detection time (min)	References
1		Xpert MTB/RIF	Semi-nested PCR	*rpoB*	F	131 CFU/mL	9.98,80(in China）	120	(9–11)
2		RealAmp	LAMP	IS*6110*	F	NR	7–8	81	(14)
3		EasyNAT TB	CPA	*gyrB*	Lateral flow test	four copies/µL	4–5	140	(16)
4		EasyNAT MTC	CPA	IS*6110*	F	< 4 copies/µL	13–14	90	(9, 12)
5		PCR	RT-PCR and FQ-PCR	IS*6110*	F	about 1000 CFU/mL	26(RT-PCR)/ 16.3(FQ-PCR）	120–240	(9)
6	AapCas12b	TB One-Pot	CPA	IS*6110*	F/visual detection under UV light	50 CFU/mL 0.8 copies/µL	1.4	80	Our method
7	Cas12a	CRISPR-MTB	RPA	IS*6110*	F	5 copies/µL; 50 CFU/mL	NR	90	(4)
8	AacCas12b	TB-QUICK	LAMP	IS*6110*	F	1.3 copies/µL (2.6 copies/reaction)	NR	120	(6)
9	Cas12a	NR	RPA	IS*1081*	F	4.48 fmol/L (about 298 copies/reaction)	NR	240	(21)
10	Cas12a	NR	RPA	IS*6110*	F	one copy//µL	NR	60	(22)
11	Cas12a	LACD	LAMP	IS*6110*	F/Lateral flow test	About 10 copies/reaction (50fg)	NR	60	(23)
12	Cas12a	CRISPR-TB	PCR	IS*6110*	F/Lateral flow test	0.25 copies/µL of purified cfDNA or 0.06 copies/µL of serum sample	NR	120	(5)
13	Cas13a	CRISPR-MTB	PCR, *in vitro* transcription	IS*1081*	F	one copy/µL	2	120	(7)
14	Cas12a	MTB-MCDA-CRISPR	MCDA	*sdaA*	F/visual detection under UV light	40 fg/reaction(eight copies/reaction）	4.5	60	(24)
15	AapCas12b	CRISPR-MCDA	MCDA	IS*6110*	F/visual detection under UV light	five fg/µL	4.5	70	(8)

^*a*^ LAMP, loop-mediated isothermal amplification; CPA, cross-priming amplification; RT-PCR, reverse transcription polymerase chain reaction; FQ-PCR, fluorescent quantitative polymerase chain reaction; RPA, recombinase polymerase amplification; MCDA, multiple cross-displacement amplification; F, fluorescent; NR, not reported.

Limitations of this study stem from the retrospective evaluation of TB One-Pot’s diagnostic performance exclusively in sputum samples. To establish more robust evidence, future investigations should encompass multicenter prospective studies that encompass larger patient cohorts and incorporate a broader spectrum of sample types for further validation of the diagnostic effectiveness of this detection method.

In this study, we created a single reaction method for TB DNA detection using CRISPR Cas12b and CPA. It is fast, highly sensitive, and cost-effective, with the potential to overcome limitations in molecular TB diagnosis, particularly in resource-limited settings.

## Data Availability

The WGS data obtained in this study have been deposited under NCBI BioProject PRJNA1019825.

## SUPPLEMENTAL MATERIAL

Fig S1 to Fig S4,Tables S1 and S2 jcm.00923-23-s0001.docx Fig. S1 (Amplification [CY5] and cas12b cleavage [FAM] fluorescence signals), S2 (TB One-Pot requires nucleic acid amplification for optimal activation, and it is most effective at higher target concentrations), S3 (Time-kinetic fluorescence signal curves of the TB One-Pot detection platform at different CFU concentrations), and S4 (Visual observation of the TB One-Pot detection platform) and Tables S1 (Results of TB One-Pot detection of 9 NTM Specimens in clinical sputum samples) and S2 (Targeted next-generation sequencing confirmed the positivity of two samples from non-TB patients detected by TB One-Pot).

ASM does not own the copyrights to Supplemental Material that may be linked to, or accessed through, an article. The authors have granted ASM a non-exclusive, world-wide license to publish the Supplemental Material files. Please contact the corresponding author directly for reuse.
