# Supplementary material for: Rapid detection of Mycobacterium tuberculosis in sputum using CRISPR-Cas12b combined with cross-priming amplification in a single reaction
Source: J Clin Microbiol. 2023 Dec 19;62(1):e00923-23. doi: 10.1128/jcm.00923-23 (PMC10793277; doi:10.1128/jcm.00923-23)
Supplement: Fig S1 to Fig S4,Tables S1 and S2 — Fig. S1 (Amplification [CY5] and cas12b cleavage [FAM] fluorescence signals), S2 (TB One-Pot requires nucleic acid amplification for optimal activation, and it is most effective at higher target concentrations), S3 (Time-kinetic fluorescence signal curves of the TB One-Pot detection platform at different CFU concentrations), and S4 (Visual observation of the TB One-Pot detection platform) and Tables S1 (Results of TB One-Pot detection of 9 NTM Specimens in clinical sputum samples) and S2 (Targeted next-generation sequencing confirmed the positivity of two samples from non-TB patients detected by TB One-Pot). [file jcm.00923-23-s0001.docx]

**Supplementary Table of Contents**

**Supplementary Figure 1 Amplification (CY5) and cas12b cleavage (FAM) fluorescence signals** **Supplementary Figure 2 TB One-Pot requires nucleic acid amplification for optimal activation, and it is most effective at higher target concentrations.**

**Supplementary Figure 3 Time-kinetic fluorescence signal curves of the TB One-Pot detection platform at different CFU concentrations.**

**Supplementary Figure 4 Visual observation of the TB One-Pot detection platform.**

**Supplementary Table 1 Results of TB One-Pot detection of 9 NTM Specimens in clinical sputum samples**

**Supplementary Table 2 Targeted next-generation sequencing confirmed the positivity of two samples from non-TB patients detected by TB One-Pot**


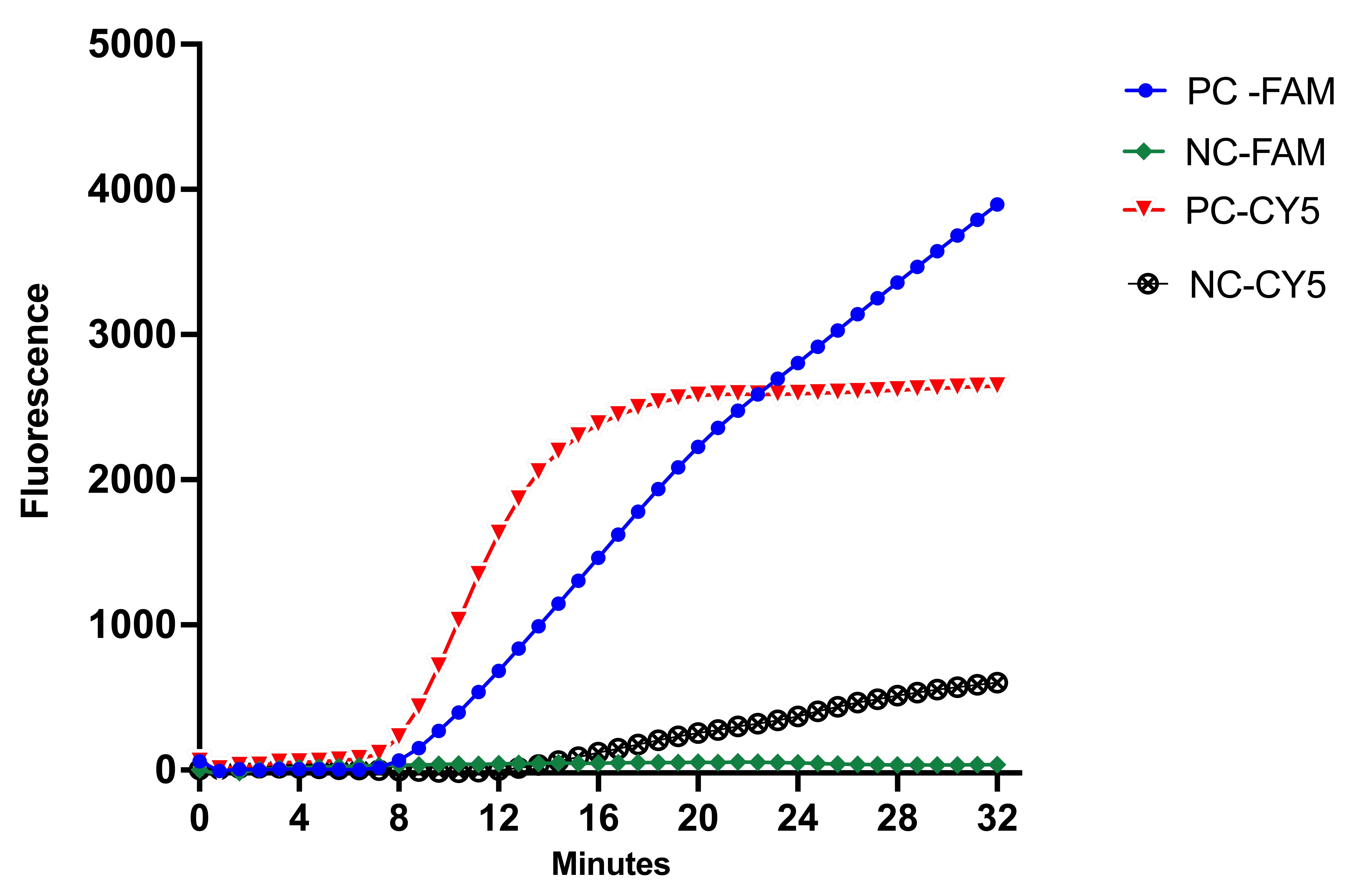


**Supplementary Figure 1 Amplification (CY5) and cas12b cleavage (FAM) fluorescence signals** (The colored line represents the mean value of 3 replicates). Positive control (PC) and negative control (NC). Incubation at 58°C for 32 minutes with fluorescence signal recorded every 48 seconds.


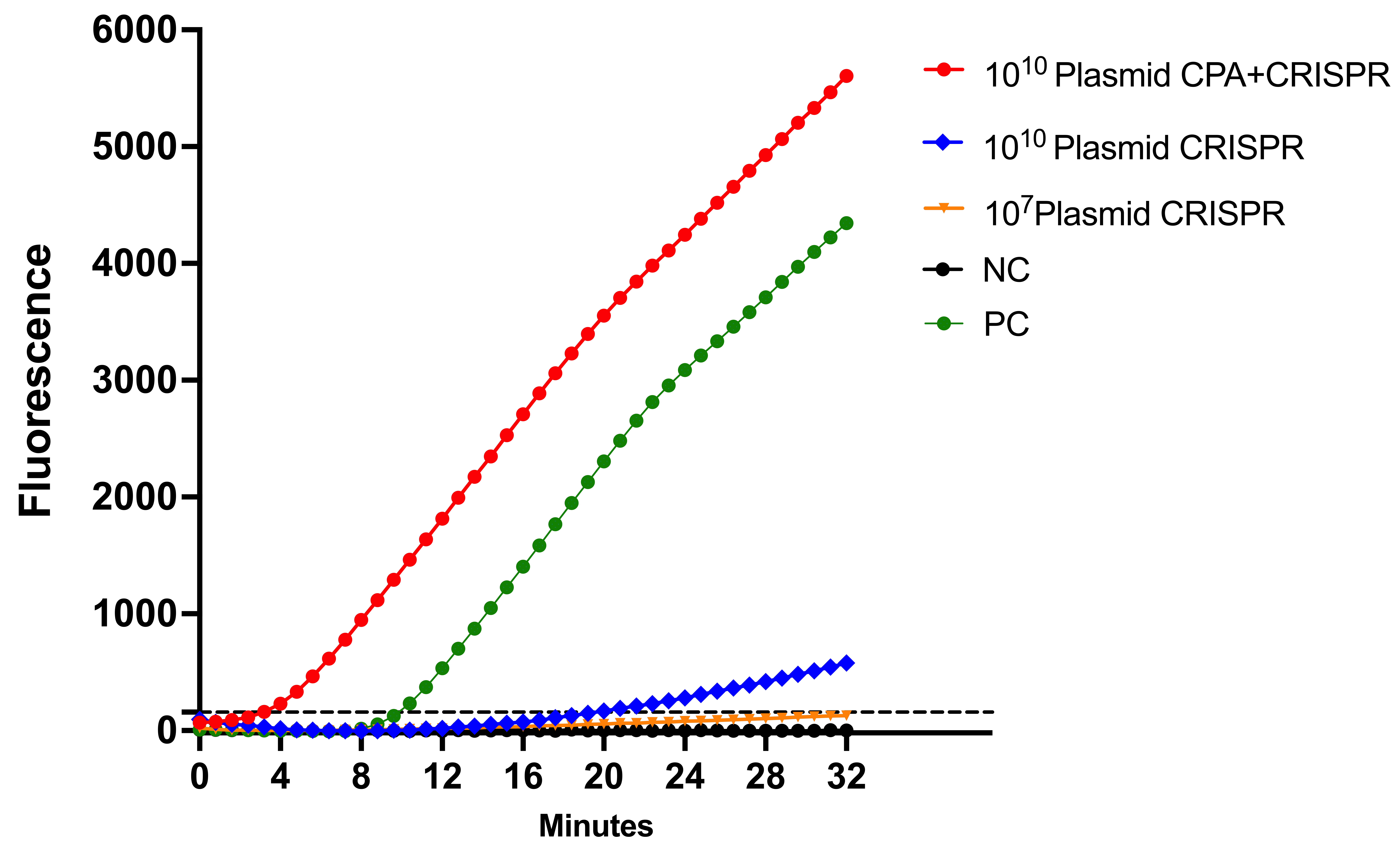


**Supplementary Figure 2 TB One-Pot requires nucleic acid amplification for optimal activation, and it is most effective at higher target concentrations.**

**

**

**Supplementary Figure 3 Time-kinetic fluorescence signal curves of the TB One-Pot detection platform at different CFU concentrations.**

**
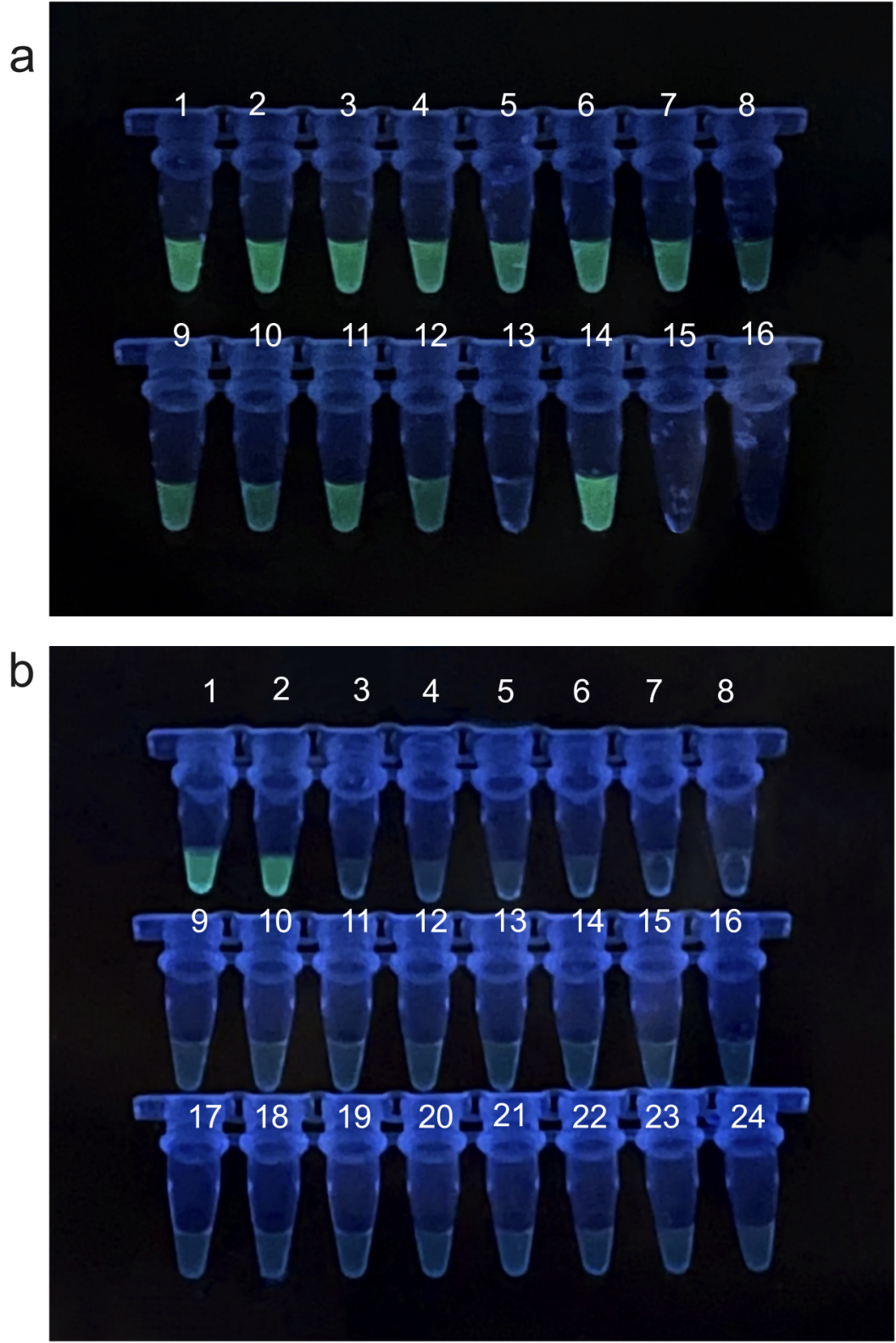
**

**Supplementary Figure 4 Visual observation of the TB One-Pot detection platform.** (a) Sensitivity of TB One-Pot. Gradient dilutions of H37Rv live bacterial suspension at a concentration of 10^8^ CFU/mL. Samples 1-12 represent concentrations of 10^7^, 10^6^, 10^5^, 10^4^, 10^3^, 500, 250, 200, 150, 100, 50, and 25 CFU/mL, respectively. Sample 13: Negative control with RNase-free water. Sample 14: Positive control with 1 ng/μL H37Rv DNA. Samples 15 and 16: Blank tubes. (b) Determination of species specificity of TB One-Pot using genomic DNA from 23 strains. Samples 1-2: H37Rv and BCG. Samples 3-23 represent *M.simiae*, *M.parascrofulaceum*, *M.avium*, *M.intracellulare*, *M.paraintracellulare*, *M.kansasii*, *M.chelonae*, *M.abscessus*, *M.triplex*, *M.lentiflavum*, *M.paragordonae*, *M.gordonae*, *M.scrofulaceum*, *M.fortuitum*, *M.colombiense*, *Aspergillus fumigatus* , *Pseudomonas aeruginosa*, *Cryptococcus neoformans*, *Escherichia coli*, *Klebsiella pneumoniae*, and *Staphylococcus aureus*. Sample 24: Negative control with RNase-free water (NC). The genomic DNA concentration of each strain was 1 ng/μL, and 5 μL was added to the reaction system. The experimental results were observed under ultraviolet light using naked-eye observation.

| **Supplementary Table 1 Results of TB One-Pot detection of 9 NTM Specimens in clinical sputum samples** | | | |
| --- | --- | --- | --- |
| clinical sample | NTM strain | Fluorescence | TB One-Pot Test Results |
| case 26 | *M.intracellulare* | <10 | Negative |
| case 32 | *M.intracellulare* | <10 | Negative |
| case 99 | *M. avium* | <10 | Negative |
| case 180 | *M. abscessus* | <10 | Negative |
| case 183 | *M.intracellulare* | <10 | Negative |
| case 198 | *M. avium* | 2605.2 | Positive |
| case 277 | *M.avium complex* | <10 | Negative |
| case 274 | *M.intracellulare* | <10 | Negative |
| case 291 | *M.intracellulare* | 10.28 | Negative |
| All Fluorescence < threshold of 157.7; NTM, non-tuberculous mycobacteria; | | | |

| **Supplementary Table 2 Targeted next-generation sequencing confirmed the positivity of two samples from non-TB patients detected by TB One-Pot** | | | | | | | | |
| --- | --- | --- | --- | --- | --- | --- | --- | --- |
| clinical sample | clinical diagnosis | barcode | Depth | Ave_Length | Ave_Score | Ave_Identity | batch_depth | batch_depth_ratio |
| Case 198 | NTM infection | IS*6110* | 1175 | 625.78 | 415.75 | 94.84 | 71148 | 1.65% |
|  |  | IS*1081* | 354 | 653.39 | 409.82 | 95.57 | 24356 | 1.45% |
| Case 241 | malignancy | IS*6110* | 7061 | 586.78 | 417.6 | 94.82 | 45962 | 15.36% |
|  |  | IS*1081* | 616 | 611.04 | 412.74 | 95.78 | 12077 | 5.10% |
| NTM, non-tuberculous mycobacteria; | | | | | | | | |
